# Progress in Neoantigen Targeted Cancer Immunotherapies

**DOI:** 10.3389/fcell.2020.00728

**Published:** 2020-07-30

**Authors:** Xue-Jiao Han, Xue-lei Ma, Li Yang, Yu-quan Wei, Yong Peng, Xia-wei Wei

**Affiliations:** Lab of Aging Research and Anticancer Target, State Key Laboratory of Biotherapy and Cancer Center, West China Hospital, Sichuan University and Collaborative Innovation Center, Chengdu, China

**Keywords:** neoantigens, cancer immunotherapy, clinical trial, checkpoint blockade, targeted cancer

## Abstract

Immunotherapies that harness the immune system to kill cancer cells have showed significant therapeutic efficacy in many human malignancies. A growing number of studies have highlighted the relevance of neoantigens in recognizing cancer cells by intrinsic T cells. Cancer neoantigens are a direct consequence of somatic mutations presenting on the surface of individual cancer cells. Neoantigens are fully cancer-specific and exempt from central tolerance. In addition, neoantigens are important targets for checkpoint blockade therapy. Recently, technological innovations have made neoantigen discovery possible in a variety of malignancies, thus providing an impetus to develop novel immunotherapies that selectively enhance T cell reactivity for the destruction of cancer cells while leaving normal tissues unharmed. In this review, we aim to introduce the methods of the identification of neoantigens, the mutational patterns of human cancers, related clinical trials, neoantigen burden and sensitivity to immune checkpoint blockade. Moreover, we focus on relevant challenges of targeting neoantigens for cancer treatment.

## Introduction

The occurrence rate of cancer is increasing rapidly (Torre et al., 2015). New methods such as immunotherapy have become an optimal choice in cancer treatment, along with chemotherapy, radiation and surgery (Restifo et al., 2012; Hamid et al., 2013; Robert et al., 2015a). The foundation of cancer immunology was based on tumor transplantation studies in syngeneic mice (Prehn and Main, 1957; Old and Boyse, 1964). Concurrently, clinicians observed that in gastric carcinoma patients, tumor-infiltration lymphocytes (TILs) were correlated with longer post-operative survival (Black et al., 1954). Recently, it has demonstrated that autologous T cells can show profound tumoricidal activity and immunotherapies based on T cells are effective in multiple human malignancies (Rosenberg, 2011; Rosenberg et al., 2011). In particular, treatment of patients with checkpoint blockade has shown a significant effect on patient survival (Hodi et al., 2010; Brahmer et al., 2012; Topalian et al., 2012; Sharma and Allison, 2015). These data provide clear evidence that endogenous T cells can recognize antigenic determinants—epitopes that present on the tumor cell surface from the major histocompatibility complexes (MHCs). Such epitopes may be originated from three classes of antigens: (1) viral antigens that are encoded by viral open reading frames (ORF) in virus-infected tumor cells (Walboomers et al., 1999; Gillison et al., 2000; Feng et al., 2008), such as hepatitis B virus (HBV) (Merlo et al., 2010), Epstein-Barr virus (EBV) (Lin et al., 2002), and human papilloma virus (HPV) (Morrow et al., 2013); (2) tumor-associated antigens (TAAs) that expression levels are very low in some normal tissues but are overexpressed in malignant cells, including oncofetal antigens, cancer testis antigens (CTA), overexpressed oncogenic proteins and selected differentiation antigens (Caballero and Chen, 2009; Melero et al., 2014; Ward et al., 2016), for example NY-ESO-1 (D’Angelo et al., 2018) and CD19 on B cell malignancies (Sabatino et al., 2016); and (3) neoantigens, which are immunogenic products of somatic mutations that are fully specific to tumors. In the late 1980s, Boon and colleagues were among the first to report that aberrant peptides derived from tumor mutations were able to elicit a tumor specific T cell response in a mouse model (De Plaen et al., 1988; Lurquin et al., 1989). A few years later, it was also observed in human tumors that somatic mutations were a source of neoantigens recognized by T cells (Coulie et al., 1995; Monach et al., 1995; Wolfel et al., 1995). Recently, studies have demonstrated that neoantigens are able to recognize cancer cells by intrinsic T cells (Lennerz et al., 2005; Castle et al., 2012; Matsushita et al., 2012; Kvistborg et al., 2014; Rajasagi et al., 2014; Wick et al., 2014; Chan et al., 2015; Cohen et al., 2015; Rizvi et al., 2015). Neoantigens are fully tumor-specific and bypass central tolerance (Heemskerk et al., 2013), and thus they are not expected to induce autoimmune toxicity and they are a potential target for cancer immunotherapy. With the recent development of cancer genomics (Garraway and Lander, 2013; Vogelstein et al., 2013) and high-throughput immunologic screening (Lin et al., 2008; Zhang et al., 2011), the goal of the analysis of neoantigens based on individual patients has become achievable, which makes neoantigen-directed immunotherapy highly attractive. In this review, we aim to introduce the framework for the identification and prioritization of neoantigens, the mutational patterns of cancer, neoantigen-related trials, mutational burden and sensitivity to immune checkpoint blockade. More importantly, we highlight the relevant challenges of targeting neoantigens for cancer treatment.

## Neoantigen Identification

**FIGURE 1:**
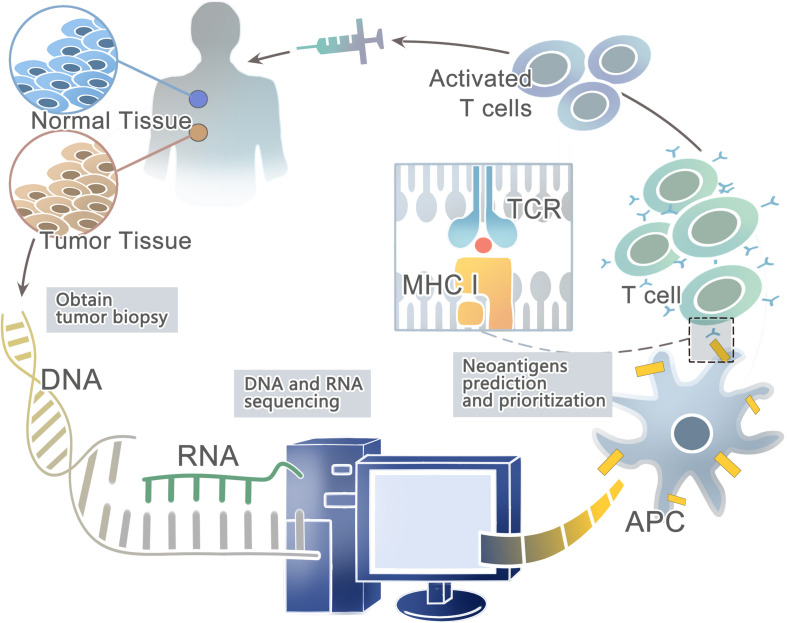
A framework for the identification and prioritization of neoantigens from computational analysis for an individual cancer sample.

Advances in next-generation sequencing (NGS) (Margulies et al., 2005; Shendure et al., 2005; Bentley et al., 2008; Wheeler et al., 2008; Drmanac et al., 2010) have allowed for the rapid and relatively inexpensive exhaustive sequencing of genomic changes across tens of thousands of human cancers (Li et al., 2011; Stratton, 2011; Gubin et al., 2015; Lu and Robbins, 2016). In conjunction, the innovation of high-throughput immunologic screening techniques has promoted the detection and isolation of neoantigens that can evoke specific T cell responses (Hadrup et al., 2009; Bentzen and Marquard, 2016; Bentzen and Hadrup, 2017) (Figure 1). In the human genome, only 1% codes for known expressed genes (the exomes) among the approximately 3 billion nucleotides (Choi et al., 2009; Ng et al., 2009). Therefore, it can significantly reduce time and costs to sequence only functional exomes. Large projects such as The Cancer Genome Atlas (TCGA) [Cancer Genome Atlas (TCGA) Research Network, 2008; Garraway and Lander, 2013] and The International Cancer Genome Consortium (ICGC) (Hudson et al., 2010) have identified cancer genomes across multiple tumor types. However, whole-exome sequencing (WES) provides limited information on non-coding regions of cancer genomes, including untranslated regions (UTRs), promoters, enhancers, introns, regulatory elements and diverse non-coding RNAs (ncRNAs) as well as unannotated regions (Garraway and Lander, 2013). In contrast, whole-genome sequencing (WGS) may be able to detect these events (Meyerson et al., 2010). Second-generation sequencing of the transcriptome (RNA-seq) is another powerful approach — as cDNA may derive from mRNA, total RNA or other RNAs, such as microRNAs and lncRNA (Morrissy et al., 2009). Recent studies have suggested that tumors harbor more abundant alternative splicing events than paired normal tissues by comprehensive analysis of WES with RNA-seq data and proteomic data, which is a potential source to generate tumor-specific neoantigens (Kahles et al., 2018; Park et al., 2018). In fact, a few studies have developed peptide arrays representing all possible frameshift peptides and detected the antibody responses to the frameshift peptides. This might be a useful method for cancer neoantigen screening (Zhang et al., 2018; Shen et al., 2019). In conclusion, somatic DNA mutations are usually computed from WGS, WES or RNA-seq data from comparisons between tumor and normal sequences (Ding et al., 2014; Tran et al., 2015).

Then the patient-specific NGS data from WGS, WES, or RNA-seq can be used to predict HLA types with computational tools such as Optiptype (Szolek et al., 2014) and Polysolver (polymorphic loci resolver) (Shukla et al., 2015). First, reads are selected from the NGS data that potentially derived from the HLA region; second, they are aligned to a full-length genomic library of all known HLA alleles (Robinson et al., 2013); and third, all the best-scoring alignments of each read are kept for further study. After predicting HLA types, computational algorithms such as NetMHC (Andreatta and Nielsen, 2016), NetMHCpan (Rammensee et al., 1999; Nielsen et al., 2007; Nielsen and Andreatta, 2016; Jurtz and Paul, 2017), and MHCflurry (O’Donnell et al., 2018) trained on large *in vitro* experimental datasets can be used to prioritize candidate neoantigens that bind to the predicted HLA types with high affinity. For example, Neopepsee and pVAC-Seq are representative analysis pipelines for tumor somatic mutations (Hundal et al., 2016; Kim et al., 2018). However, these algorithms are not a good predictor of actual HLA presentation (Bassani-Sternberg et al., 2016; Abelin et al., 2017), with only <5% of predicted peptides found on tumor cell surface (Yadav et al., 2014; Bassani-Sternberg et al., 2015). On the other hand, these prediction strategies do not consider proximal variants that can alter peptide sequences and affect neoantigen binding predictions (Hundal and Kiwala, 2019). Recently, a new prediction model-EDGE based on tumor HLA peptide mass spectrometry (MS) datasets has increased the positive predictive value up to nine-fold. However, it still does not incorporate TCR binding or predict HLA class II binding epitopes (Bulik-Sullivan et al., 2018).

**FIGURE 2:**
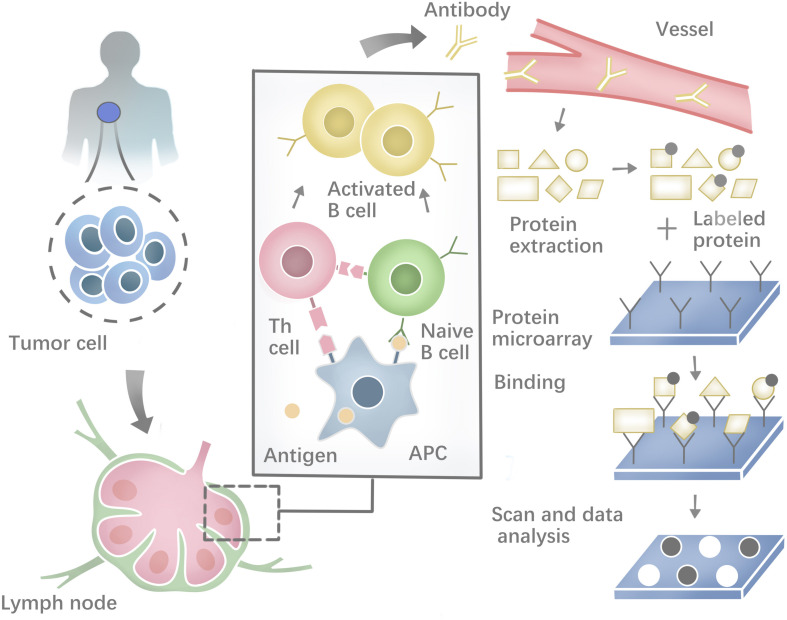
Profiling the humoral immune response and the process of protein microarray to capture autoantibodies against antigens.

Besides, a range of methodologies can be used to identify autoantibodies against tumor neoantigens based on B-cell response. The costimulatory molecules from CD4+ helper T cells and the neoepitopes presented on the surface of antigen-presenting cells (APCs) could activate the Naïve B cells in lymphoid organs. Most activated B cells will then differentiate into plasma cells to produce antibodies against tumor neoantigens (Zaenker et al., 2016). Protein microarrays are time-effective high-throughput tools (Figure 2). Sandwich immunoassays in the miniaturized system could successfully identify tumor antigens in sera samples extracted from patients (Pollard et al., 2007; Yang et al., 2013). First described by O’Farrell (1975), another tool named Serologic Proteome analysis (SERPA) or 2-D western blots, consists of the isoelectric focusing (IEF) gel run in the first dimension and SDS-PAGE gel run in the second dimension. SERPA separates the proteins in the gel by their isoelectric point (IP) and molecular mass and then transfers the proteins from the gel to a carrier membrane to screen antibodies. Finally, the antigenic protein spots can be identified by MS (Tjalsma et al., 2008). This approach has been used to identify antigens in different tumor types (Dai et al., 2017; Belousov et al., 2019). Serological analysis of recombinant cDNA expression libraries (SEREX), which combines serological analysis with antigen cloning techniques, is a widely used technique to explore tumors’ antigen repertoire. SEREX first construct a cDNA library from cancer cell lines or fresh tumor samples, then screen the cDNA library with autologous sera of cancer patients, and finally sequence the immune-reactive clones. Despite the laborious process, SEREX have identified a variety of tumor antigens including CTAs, differentiation antigens, mutational antigens, splice-variant antigens and over-expressed antigens (Chen et al., 1997; Scanlan et al., 1998; Türeci et al., 1998a, b; Brass et al., 1999; Jäger et al., 1999). Furthermore, other methods such as Multiple Affinity Protein Profiling (MAPPing) and nanoplasmonic biosensor have also been developed to identify tumor antigens (Lee et al., 2015; Jo et al., 2016).

## Mutational Patterns in Human Cancers

Somatic mutations presenting in cancer cells but not in a patient’s germ line are the primary cause of cancers (Weir et al., 2004). The cancer genome alterations include single base substitutions, insertion-deletions (so-called indels), rearrangements (inversions, translocations, duplications, and deletions) (Stratton et al., 2009; Alexandrov et al., 2013; Barrick and Lenski, 2013; Vogelstein et al., 2013; Helleday et al., 2014), but also new DNA sequences acquired from exogenous sources, notably those of viruses such as HBV, HPV, and EBV (Talbot and Crawford, 2004). Somatic mutations can be classified as driver and passenger mutations, and driver mutations, such as BRAF, KRAS, EGFR, IDH1, and PIK3CA, can provide a selective growth advantage and promote cancer development while passenger mutations do not (Haber and Settleman, 2007). Even though cancer has a mere handful of driver mutations, they are still attractive targets for immunotherapy when they are shared between different cancers and individuals. The rates of mutations vary among tumors and cancer types. Some cancer types, such as medulloblastomas, carcinoids, acute leukemias, and testicular germ cell tumors, generally carry relatively few mutations (Greenman et al., 2007; Parsons et al., 2011). However, lung cancers and melanomas occasionally have more than 100,000 mutations (Ding et al., 2008; Kan et al., 2010; Pleasance et al., 2010a), most likely because of overwhelming exposure to mutagenic carcinogen such as ultraviolet (UV) light (Trucco et al., 2018) and tobacco carcinogens (Pleasance et al., 2010b). Except for exogenous mutagenic exposure, endogenous mutational processes, such as mismatch repair deficiency in some colorectal cancers (Pena-Diaz et al., 2017) or upregulation of APOBEC cytosine deaminases, can also contribute to mutation burden (Alexandrov et al., 2013; Helleday et al., 2014). More information about the somatic mutations in human cancer can be found in COSMIC, the Catalog Of Somatic Mutations In Cancer^1^ (Forbes et al., 2015). One can even explore how cancer mutations impact the structure and function of more than 8,000 human proteins in COSMIC-3D (No authors, 2017a). Non-synonymous somatic mutations, which can alter amino acid coding sequences, are the main cause of neo-epitopes (Wood et al., 2007). Here, we summarize the number of non-synonymous somatic mutations in different cancer types (Table 1). For example, Sjoblom et al. (2006) identified 1,307 somatic mutations among 13,023 genes in 11 breast and 11 colorectal cancers (83% were missense mutations, 6% were nonsense, and the remainder were insertions, deletions, duplications, and changes in non-coding regions). Neoantigens generated from tumor somatic DNA mutations can be identified by the approach described in the first section.

**TABLE 1:** Non-synonymous somatic mutations in different cancers.

**Cancer types**	**Non-synonymous somatic mutations**	**References**
Acute myeloid leukemia	10, *n* = 1	Ley et al., 2008
Breast cancer	Average of 84, *n* = 11	Sjoblom et al., 2006; Wood et al., 2007
Glioblastoma	Average of 36, *n* = 21	Parsons et al., 2008
Pancreatic cancer	Average of 48, *n* = 24	Jones et al., 2008
Hepatocellular cancer	63, *n* = 1	Totoki et al., 2011
Colon cancer	Average of 76, *n* = 11	Sjoblom et al., 2006; Segal et al., 2008
Melanoma	Average of 201, *n* = 14	Wei et al., 2011
Lung cancer	More than 300, *n* = 1	Lee et al., 2010

Except for somatic non-synonymous protein-altering mutations, tumor neoantigens can be generated from alternative splicing variations. Pre-mRNA splicing is a biological process that contains the removal of introns and the ligation of exons to form mature RNA products (Sharp, 1994). However, the splice site and exons are alternative, which means a single gene can produce numerous mRNA isoforms (Nilsen and Graveley, 2010). And genomic variants in splicing regulatory sequences can disrupt splicing and produce aberrant mRNA and protein products (Pagani and Baralle, 2004). Recently, studies have suggested that tumors harbor more abundant alternative splicing events than paired normal tissues by comprehensive analysis of WES with RNA-seq data and proteomic data. For example, Kahles et al. (2018) performed a comprehensive analysis of alternative splicing across 32 tumor types from 8,705 patients and they found that tumors have on average 20% more alternative splicing events than normal tissues. They mainly focused on five types of alternative splicing events including exon skipping, mutually exclusive exons, intron retention, as well as alternative 3′ and alternative 5′ splice site changes. What’s more, they found that tumors harbored more exon–exon junctions termed neojunctions than normal tissues. Besides, they confirmed neojunctions-derived peptides from the Clinical Proteomic Tumor Analysis Consortium (CPTAC) and predicted the underlying target, MHC-I. The evidence showed that neojunctions-derived peptides were potential neoantigens for cancer immunotherapy. Multiple computational methods and databases have been developed to identify alternative splicing events from RNA-seq data, such as SplAdder and CancerSplicingQTL^2^ (Kahles et al., 2016; Tian et al., 2019). Smart and Margolis (2018) developed a computational strategy to identify neoepitopes generated from intron retention events in tumor transcriptomes and confirmed that these neoepitopes were processed and presented on MHC-I. Their results suggested that RNA-derived neoantigens were promising candidates for a personalized cancer vaccine (Smart and Margolis, 2018). Another mechanism to promote proteome diversity is RNA editing, which will influence RNA metabolism and function. The most prevalent form of RNA editing is the deamination of adenosine (A) to inosine (I) which may be a source for neoantigen production (Peng et al., 2018). What’s more, databases of A-to-I RNA editing events in humans have been developed, such as REDIportal^3^ and RADAR^4^ (Ramaswami and Li, 2014; Picardi et al., 2017). Recently, Zhang and Fritsche (2018) demonstrated that epitopes derived from RNA editing were presented by HLA molecules and capable of inducing immune responses.

## Neoantigen Clinical Trial

Promising results of neoantigen vaccines from preclinical studies have raised significant interest in clinical development (Castle et al., 2012; Gubin et al., 2014; Kreiter et al., 2015; Zhang et al., 2017). Recently, the clinical response in patients with advanced melanoma who received neoantigen vaccines treatment is quite encouraging in several phase I clinical trials (Yadav et al., 2014; Ott et al., 2017; Sahin et al., 2017). The main platforms for neoantigen vaccine are synthetic long peptide (SLP) vaccine, DNA vaccine, RNA vaccine, and dendritic cell (DC) vaccine. In addition, adoptive T cell therapy (ACT) targeting neo-epitopes has shown significant efficacy. Currently, there is an increasing number of clinical trials on neoantigen vaccines in a variety of cancers (Tables 2, 3).

**TABLE 2:** Ongoing clinical trials targeting cancer neoantigens.

**ClinicalTrial.gov identifier**	**Phase**	**Enrollment status**	**Sample size**	**Vaccine**	**Cancer type**	**Primary endpoint**
NCT03645148	I	Recruiting	20	Biological: iNeo-Vac-P01 Other: GM-CSF	Pancreatic cancer	(1) Objective response rate; (2) Number of participants experiencing clinical and laboratory adverse events
NCT02950766	I	Recruiting	20	Biological: NeoVax	Kidney cancer	Number of participants with dose-limiting toxicity experienced within 49 days of treatment initiation as assessed by CTCAE v4.0
NCT03558945	I	Recruiting	60	Biological: Personalized neoantigen vaccine	Pancreatic cancer	Overall time: the time between operation and the death of patients
NCT03662815	I	Active, not recruiting	30	Biological: iNeo-Vac-P01 Other: GM-CSF	AMST	(1) Objective response rate; (2) Number of participants experiencing clinical and laboratory adverse events
NCT03122106	I	Active, not recruiting	15	Biological: Personalized neoantigen DNA vaccine	Pancreatic cancer	Safety of neoantigen DNA vaccine as measured by the number of subjects experiencing each type of adverse event
NCT03532217	I	Recruiting	20	Biological: Neoantigen DNA vaccine Other: PROSTVAC-V PROSTVAC-F	Metastatic hormone-sensitive prostate cancer	(1) Safety and tolerability of regimen as defined by incidence of adverse events; (2) Immune response as measured by tetramers; (3) Immune response as measured by genomic studies; (4) Immune response as measured by flow cytometry; (5) Safety and tolerability of regimen as defined by incidence of dose-limiting toxicities
NCT02632019	I, II	Unknown status	40	Biological: Dendritic cell-precision T cell for neo-antigen	Advanced biliary tract malignant tumor	Overall survival
NCT03199040	I	Recruiting	24	Biological: Neoantigen DNA vaccine	Triple negative BC	Safety of neoantigen DNA vaccines given alone or in combination with Durvalumab as measured by number of adverse events experienced by patient
NCT03639714	I, II	Recruiting	214	Biological: GRT-C901 GRT-R902	NSCLC; CC; Gastroesophageal; Adenocarcinoma; Urothelial Carcinoma	(1) Incidence of adverse events, serious adverse events, and dose-limiting toxicities; (2) Objective response rate in phase 2 using RECIST v1.1; (3) Identify the recommended Phase 2 dose of GRT-C901 and GRT-R902
NCT03606967	II	Not yet recruiting	70	Biological: Personalized synthetic long peptide vaccine Others: Poly ICLC	Anatomic stage IV BC	Progression-free survival
NCT03715985	I	Recruiting	25	Biological: EVAX-01-CAF09b	Malignant melanoma metastatic; NSCLC metastatic; Kidney cancer metastatic	Number and type of reported adverse events
NCT03412877	II	Recruiting	210	Biological: Individual patient TCR-transduced PBL	Glioblastoma; NSCLC; Ovarian Cancer; BC; Gastrointestinal/Genitourinary cancer	Response rate
NCT03171220	I, II	Recruiting	40	Biological: Neoantigen reactive T cells (NRTs) Other: SHR-1210	AMST	Number of participants with adverse events
NCT03359239	I	Recruiting	15	Biological: PGV001 Other: Poly ICLC	Urothelial/Bladder cancer; Nos	(1) Number of neo-antigens; (2) Number of peptides synthesized; (3) Vaccine Production time; (4) Proportion of consent to tissue acquisition phase; (5) Proportion of subjects eligible for the treatment phase; (6) Number of toxicities
NCT03658785	I, II	Not yet recruiting	40	Biological: TIL	RC; MC; Solid tumor	Objective response rate
NCT03674073	I	Recruiting	24	Biological: Neoantigen vaccines	HCC	Safety of neoantigen-based DC vaccine as measured by the number of subjects experiencing each type of adverse event according to the National Cancer Institute Common Terminology Criteria for Adverse Events v4.0.
NCT01970358	I	Active, not recruiting	20	Biological: Peptides Other: Poly-ICLC	Melanoma	(1) Number of participants experiencing clinical and laboratory adverse events; (2) Number of participants for whom sequencing and analysis leads to identification of at least 10 actionable peptides to initiate vaccine production
NCT02287428	I	Active, not recruiting	46	Biological: Personalized neoantigen vaccine	Glioblastoma	(1) Cohorts 1, 1a, 1b, and 1c: Number of participants with adverse events as a measure of safety and tolerability; (2) Cohorts 1d: Number of participants with adverse events as a measure of safety and tolerability; (3) Cohort 1: Number of participants with at least 10 actionable peptides as a measure of study feasibility; (4) Cohort 1: Number of participants who are clinically able to initiate post-RT vaccine therapy within 12 weeks or less from date of surgery
NCT03361852	I	Not yet recruiting	20	Biological: NeoVax	Follicular lymphoma	Feasibility of Neovax following 4 weekly doses of Rituximab
NCT03219450	I	Not yet recruiting	10	Biological: NeoVax	Lymphocytic leukemia	(1) The proportion of all enrolled patients for whom sequencing and analysis leads to identification of at least 7 actionable peptides to initiate vaccine production; (2) The proportion for whom the time from sample collection to vaccine availability is less than 12 weeks; (3) The number of patients with treatment-limiting toxicities

*All the relevant information of clinical trials was registered on the official website (clinicaltrials.gov). AMST (advanced malignant solid tumor), BC (breast cancer), NSCLC (non-small cell lung cancer), CC (colorectal cancer), HCC (hepatocellular carcinoma), RC (recurrent cancer), MC (metastatic cancer).*

**TABLE 3:** Completed clinical trials targeting cancer neoantigens.

**ClinicalTrial.gov identifier**	**Vaccine**	**Phase**	**Cancer type**	**Mechanism**	**Primary outcome**	**Adverse events**	**References**
NCT00683670	DC vaccine	I	Melanoma	Autologous DC vaccines directed at tumor amino acid substitutions	Vaccination broadened the antigenic breadth and clonal diversity of anti-tumor immunity	–	Carreno et al., 2015
NCT01970358	Peptide vaccine	I	Melanoma	Vaccine targeting up to 20 predicted personal tumor neoantigens	Four of six vaccinated patients experienced no recurrence and another two relapse patients experienced CR to anti- PD-1 therapy	Fatigue, rash, injection site reactions, mild flu-like symptoms	Ott et al., 2017
NCT01174121	TIL vaccine	II	Epithelial cancer	Mutated ERBB2IP-specific CD4^+^ T cell response	The patient experienced disease stabilization for about 13 months after cell infusion	–	Tran et al., 2014
NCT00204607	RNA vaccine	I/II	Malignant melanoma	Protamine-stabilized mRNA vaccine coding for Melan-A, Tyrosinase, gp100, Mage-A1, Mage-A3, and Survivin	Vaccine-directed T cells increased, while the frequency of immunosuppressive cells decreased. No adverse events more than grade II were observed	Injection site reactions, fatigue and flu-like symptoms	Weide et al., 2009
NCT02287428	Peptide vaccine	I	Glioblastoma	Vaccines contained up to 20 long peptides that were divided into pools of 3–5 peptides admixed with poly-ICLC	Median PFS and OS were 7.6 and 16.8 months, respectively	Chills, dizziness, fatigue, flushing, headache, myalgia, nausea and injection site reaction	Keskin et al., 2019
NCT02035956	RNA vaccine	I	Melanoma	RNA vaccine encoding shared tumor-associated self-antigens	Two patients had a vaccine-related OR among the five patients with metastatic disease, and the other eight patients mostly experienced prolonged DFS	–	Sahin et al., 2017
NCT01174121	TIL vaccine	I	Metastatic colorectal cancer	TIL vaccine that specifically targeted KRAS G12D	The objective regression of six lung metastases was observed, while one metastatic lesions had progressed	-	Tran et al., 2016

*DC (dendritic cell), TIL (tumor-infiltrating lymphocyte), CR (complete responses), PFS (progression-free survival), OS (overall survival), OR (objective response), DFS (disease-free survival).*

### SLP Vaccine

The peptide vaccine is fast and flexible, and it is simple to make an individual cocktail for patients. Antigenic peptide has been widely developed in vaccination due to its strengths including low cost, low toxicity and direct function as pivotal T cell epitope (Li et al., 2014; Kumai et al., 2017). Ott et al. (2017) developed an immunogenic personal neoantigen vaccine (NCT01970358). In this phase I clinical study, 6 patients with resected high-risk melanoma (stage IIIB/C and IVM1a/b) received personalized long peptide vaccines targeting up to 20 neoantigens per patient after sequencing and prioritizing HLA class I prediction (toll-like receptor 3 [TLR3] and melanoma differentiation-associated protein 5 [MDA-5] poly-ICLC were also co-administered as adjuvants). The vaccination activated both CD8+ and CD4+ T cells responses against tumor. Treatment-related adverse events were fatigue, rash, injection site reactions and mild flu-like symptoms, with no major autoimmune toxicity. Encouragingly, four of these six vaccinated patients experienced no recurrence at 25 months after vaccination, and another two relapse patients with metastatic disease later experienced complete responses to anti- PD-1 therapy (Ott et al., 2017). However, the therapeutic efficacy of SLP vaccine is limited by inefficient delivery to desired lymphoid organs as it could rapidly diffuse into the peripheral blood vessels due to its small molecular size (Irvine et al., 2015; Zhu and Zhang, 2017).

### DNA Vaccine

DNA vaccine is stable, safe in handling, cost-efficient and easy to manufacture (Prazeres and Monteiro, 2014). Importantly, DNA vaccine can activate both CD4+ and CD8+ T cell response, as well as innate immune response due to the recognition of double stranded DNA structure by cytosolic sensors (Tang and Pietersz, 2009; Ori et al., 2017). Newer DNA vaccines have shown efficacy in the clinic (Trimble et al., 2015; Tebas et al., 2017; Aggarwal et al., 2019). Recently, one group utilized a DNA vaccine platform to target tumor neoantigens in a mouse model. They chose TC1, LLC, and ID8 as tumor models that hardly respond to immune-checkpoint blockade alone. After sequencing these tumors and identifying neoantigens, they designed 12 epitopes per plasmid and these synthetic neoantigen DNA vaccines (SNDVs) were tested *in vivo*. Not surprisingly, it showed robust T cell immunity. Intriguingly, a larger proportion of CD8+ T cell responses was generated during the SNDVs treatment (25% CD4+ and 75% CD8+ T cell responses) compared with other platforms such as SLP neoantigen vaccines (60% CD4+ and 16% CD8+ T cell responses). Although the epitopes were selected *in silico* for high MHC class I binding affinity, SLP and RNA neoantigen vaccines generated a higher proportion of MHC class II–restricted CD4+ T cells (Kreiter et al., 2015; Martin et al., 2016; Ott et al., 2017; Sahin et al., 2017). However, DNA vaccine shows poor immunogenicity in human trials (Jorritsma et al., 2016).

### RNA Vaccine

The advantages of RNA vaccine include low risk of insertional mutagenesis, direct translation into the cytoplasm and simple and inexpensive manufacturing procedure (Sahin et al., 2014). Kreiter et al. (2015) developed synthetic poly-neo-epitope messenger RNA vaccines after exome sequencing and bioinformatic prioritization in three independent murine tumor models. This vaccination induced complete rejection of established tumors and reshaped the tumor microenvironment (Kreiter et al., 2015). As RNA is the genetic material in many viruses, the human immune system tends to be on high alert for it, which gives an RNA vaccine a unique advantage. “It is its own adjuvant,” Sahin says (Sahin et al., 2017), so he implemented the RNA-based poly-neo-epitope vaccines in 13 patients with stage III-IV melanoma (NCT02035956). Two patients had a vaccine-related objective response among the five patients with metastatic disease, and the other eight patients who had no detectable disease mostly experienced prolonged disease-free survival. Two-thirds of vaccination developed *de novo* in addition to pre-existing immunity. Weide et al. (2009) also showed that direct injection of protamine-protected mRNA vaccine is feasible and safe; it can also increase the T cell response and decrease immunosuppressive cells in metastatic melanoma patients (NCT00204607). However, the translational efficiency of RNA vaccine remains challenging as only a small portion of administered mRNA can be captured and presented by APCs. Therefore, Sahin and his group administered the RNA vaccine directly into lymph nodes through ultrasound-guided percutaneous injection, noted as intranodal injection (i.n.).

### DC Vaccine

Dendritic cells have a key role in presenting antigens to the immune system. DCs are often recognized as the most potent APCs, which are capable of acquiring and processing antigens for presentation to T cells and expressing high levels of costimulatory molecules (Sabado et al., 2017). Therefore, vaccination based on DCs is a promising platform for neoantigen vaccine. Carreno et al. (2015) were the first to report autologous DC vaccines directed at tumor amino acid substitutions (AAS) in three melanoma patients (NCT00683670). They filtered the candidate HLA-A^∗^ 02: 01 epitopes containing mutations residues after whole exome sequencing and HLA binding prediction and evaluated the MHC-epitope binding using mass spectrometry. Then they filtered precursors of DCs from patients’ bloodstream, matured them and exposed them to synthetic epitopes. The peptide-loaded DCs were then returned to the patients by intravenous infusion. It increased the breadth and diversity of anti-tumor immunity after receiving the DC neoantigen vaccine (Carreno et al., 2015). However, DC vaccine is laborious, costly and need highly skilled technicians for manufacturing (Chen et al., 2016).

### Adoptive T Cell Therapy (ACT)

T cell therapy targeting driver mutations is quite attractive, since they are not only specific and biologically important, but also shared between different patients (McGranahan et al., 2015). Currently, KRAS mutations are hot-spot driver mutations and the most frequent KRAS mutant is KRAS G12D that is expressed in ∼45% of pancreatic adenocarcinomas (Bryant et al., 2014) and ∼13% of colorectal cancers (Vaughn et al., 2011). Rosenberg et al. (2016) administered cytotoxic T cells targeting mutant KRAS G12D into a patient with metastatic colorectal cancer (NCT01174121). After whole-exome and transcriptome sequencing of three resected lung lesions, they found that CD8+ T cells in TILs specifically recognized mutant KRAS G12D. Then they selected and expanded TILs that were reactive to the mutant KRAS G12D. The patient received a single infusion of 1.48 × 10^11^ TILs, which contained 1.11 × 10^11^ HLA-C^∗^08:02–restricted CD8+ T cells that specifically targeted KRAS G12D. The objective regression of all seven lung metastases was observed at the first follow-up visit. However, one of these metastatic lesions had progressed when evaluating after 9 months of therapy. The loss of the chromosome 6 haplotype encoding the HLA-C^∗^08:02 class I MHC molecule resulted in progression after resecting this lesion and sequencing, which provides direct evidence of tumor immune evasion (Tran et al., 2016). Furthermore, the group used this approach to demonstrate that CD4+ T helper 1 (TH1) cells in TILs recognized a mutation in erbb2 interacting protein (ERBB2IP) in a patient with metastatic cholangiocarcinoma. This patient was treated with mutation-reactive TH1 cells twice and experienced tumor regression (NCT01174121) (Tran et al., 2014).

## Neoantigen Load Associates With Immune Checkpoint Inhibitors

Antibodies targeting two immune checkpoints, PD-1 and CTLA-4, represent the greatest success of cancer immunotherapy, which can elicit durable antitumor responses in a wide range of malignancies (Hodi et al., 2010; Hamid et al., 2013; Chen and Han, 2015; Postow et al., 2015; Robert et al., 2015a; Zou et al., 2016). The treatment of immune checkpoint inhibitors has improved OS and PFS in many different cancers (Robert et al., 2011; Topalian et al., 2014; Borghaei et al., 2015; Brahmer et al., 2015; Garon et al., 2015; Larkin et al., 2015; Robert et al., 2015b; Sundar et al., 2015; George et al., 2016; Tomita et al., 2017). Accumulating evidence suggests that cancers with higher mutation burden are associated with more survival benefits from both anti- PD-1 and anti- CTLA-4 therapy (Table 4) (Hamid et al., 2013; Asaoka et al., 2015; Rizvi et al., 2015; Andor and Graham, 2016; Erratum for the Report “Genomic correlates of response to CTLA-4 blockade in metastatic melanoma” by Van Allen et al., 2016; Hugo et al., 2016; Matsushita et al., 2016; McGranahan et al., 2016; Morris et al., 2016; Rosenberg et al., 2016). Mutations may increase the possibility of generating immunogenic neoantigens, which facilitate the recognition of cancer cells as foreign (Schumacher and Schreiber, 2015; Riaz and Havel, 2016). Studies in melanoma patients have demonstrated that neoantigen-specific CD8+ and CD4+ T cells in TILs responded to checkpoint blockade therapy (Kvistborg et al., 2014; Lu et al., 2014; Tran et al., 2014; Linnemann et al., 2015), which provides testimony for the hypothesis. Furthermore, neoantigen loss may contribute to acquired resistance through tumor cell elimination or chromosomal deletions during immune checkpoint blockade therapy (Anagnostou et al., 2017); other mechanisms include upregulation of alternate immune checkpoints (Koyama et al., 2016), loss of HLA haplotypes (Maeurer et al., 1996), or somatic mutations in HLA or JAK1/JAK2 genes (Shukla et al., 2015; Garcia-Diaz et al., 2017). However, a proof-of-concept by Nicholas in which cytotoxic chemotherapy–induced subclonal neoantigens were enriched in certain poor responders to immune checkpoint inhibitors was presented (McGranahan et al., 2016). Additionally, gliomas that recurred after treatment with the DNA alkylating agent temozolomide were identified to carry numbers of mutations (Hunter et al., 2006; Cahill et al., 2007), which had a higher mutation burden generated from chemotherapy, but had less clinical benefit. As a result, the association between response to immune checkpoint blockade and neoantigen burden is not linear and clear (Le et al., 2015). Matsushita et al. (2017) demonstrated that the number of neoantigens per missense mutation (neoAg frequency) was an independent predictive factor for PFS in ovarian clear cell carcinoma (OCCC), and the low neoAg frequencies were correlated with increased PFS. High mutation and neoantigen load negatively influenced PFS in multiple myeloma (MM) patients, which had a lower mutational burden (Miller et al., 2017). Luksza et al. (2017) developed a neoantigen fitness model that could describe the evolutionary dynamics of cancer cells and predict tumor response to checkpoint blockade immunotherapy. Further studies are needed regarding the relationship between neoantigen load and checkpoint blockade immunotherapy.

**TABLE 4:** Trials defining a TMB threshold for ICB benefit.

**Cancer type**	**Agents**	**Methods**	**Threshold defined**	**RR**	**PFS**	**References**
NSCLC	Pembrolizumab	WES	200 mut	59% versus 12%	NR versus 3.4 months	Rizvi et al., 2015
NSCLC	Anti-PD-1 and anti-PD-L1	MSKCC and NGS	7.4 mut/Mb	38.6% versus 25%	–	Rizvi et al., 2018
NSCLC	Nivolumab and ipilimumab	WES	158 mut	51% versus 13%	17.1 versus 3.7 months	Hellmann et al., 2018a
SCLC	Nivolumab and ipilimumab	WES	248 mut	46.2% versus 21.3%	7.8 versus 1.4 months	Hellmann et al., 2018b
NSCLC	Nivolumab	WES	>243 mut	47%versus 23%	HR 0.62	Carbone et al., 2017
NSCLC	Nivolumab and ipilimumab	FM and NGS	>10 mut/Mb	45.3% versus 24.6%	7.1 versus 3.2 months	Hellmann et al., 2018c
Melanoma	Anti-CTLA-4	WES	100 mut	–	–	Snyder et al., 2014
Melanoma	Nivolumab	WES	100 mut	–	–	Riaz et al., 2017
Various cancers	Various immunotherapies	FM and NGS	20 mut/Mb	58% versus 20%	12.8 versus 3.3 months	Goodman et al., 2017
UC	Atezolizumab	FM and NGS	16 mut/Mb	–	–	Balar et al., 2017
UC	Atezolizumab	FM and NGS	>9.65 mut/Mb	–	–	Powles et al., 2018
UC	Nivolumab	WES	≥170 versus < 85 mut	31.9% versus 10.9%	3 versus 2 months	Galsky et al., 2020

*RR (relative risk), PFS (progression-free survival), NSCLC (non-small-cell lung cancer), SCLC (small-cell lung cancer), UC (urothelial cancer), PD-1 (programmed cell death protein 1), PD-L1 (programmed cell death-ligand 1), CTLA-4 (cytotoxic T-lymphocyte-associated protein 4), WES (whole exome sequencing), NGS (next generation sequencing), FM (foundation medicine), mut (mutation), HR (hazard ratio).*

High mutation burden is associated with survival benefits from ICB therapy, but autoantibodies may also predict the efficacy of immune checkpoint inhibitors (ICIs) (De Moel et al., 2019). However, recent studies have demonstrated that autoantibodies correlate with immune checkpoint therapy-induced toxicities (Da Gama et al., 2018; De Moel et al., 2019; Tahir et al., 2019). Since the mechanism of ICB therapy is to mediate non-specific suppression of T cells by negative costimulatory signals, it may break the balance of autoimmunity and lead to the activation of autoreactive B cells that produce autoantibodies (Pardoll, 2012; Oh et al., 2017). As we know, ICB therapy can cause immune-related adverse events (irAEs), referring to the release of distinctive toxicities including pneumonitis, dermatitis, hepatitis, colitis, and hypophysitis (Hodi et al., 2010; Weber et al., 2012; Gao et al., 2015). A recent study found that CD21lo B cells and plasmablasts increased in patients following ICB treatment, and these changes in B cells preceded and correlated with the frequency and timing of irAEs (Das et al., 2018). Besides, one phase I clinical trial including advanced metastatic melanoma patients who received BCG and ipilimumab treatment was suspended due to the occurrence of irAEs. Researchers found profound increases in the repertoire of autoantibodies directed against both selves- and cancer antigens at time points preceded the development of symptomatic toxicity (NCT01838200) (Da Gama et al., 2018). Furthermore, Tahir et al. (2019) also found that anti-GNAL and anti-ITM2B autoantibodies correlated with the development of ICI–related hypophysitis and that anti-CD74 autoantibodies were associated with ICB–induced pneumonitis development. They also tested additional patient samples by enzyme-linked immunosorbent assay to verify these findings (Tahir et al., 2019). These data suggest that autoantibodies under ICB treatment may serve as a predictive biomarker for irAEs.

## Challenges

### The Challenges With MHC–Peptide-Binding Prediction

Current neoantigen identification techniques are still time consuming and labourious (Tran et al., 2015; Gros et al., 2016). The predictors of immunogenicity are immature (Calis et al., 2013). In addition, since cytotoxic CD8+ T cells are the main killer of cancer cells, the available computational tools can only predict neo-epitopes that bind to MHC class I molecules presented on CD8+ T cells (Nielsen and Andreatta, 2016). Humans have approximately 5,000 alleles encoding MHC class I molecules, with expression of up to six MHC class I molecules (No authors, 2017b) and computational tools cannot predict them all. Intriguingly, even though the epitopes are selected for high MHC class I binding affinity, the neoantigen vaccine trials showed a higher proportion of MHC class II–restricted, CD4+ T cells (Kreiter et al., 2015; Ott et al., 2017). Furthermore, studies demonstrated that CD4+ T cells also recognize a higher number of neo-epitopes than was previously known and can confer potent antitumor activity (Tran et al., 2014; Kreiter et al., 2015). However, it will be more difficult to develop predictive algorithms for MHC class II molecules (Nielsen et al., 2010). First, MHC class II molecules are heterodimers of alpha and beta peptides encoded by four different loci, with three of them being highly polymorphic in the human genome (Robinson et al., 2003). Second, the MHC class II binding groove is open on both ends, presenting longer sequences of amino acids (11–20 amino acids or even longer) than MHC class I molecules (8–11 amino acids) (Babbitt et al., 1985; Bjorkman et al., 1987). Recently, Andreatta et al. (2015) described the method for the quantitative prediction of peptide binding affinity of MHC class II molecules of known sequence.

**FIGURE 3:**
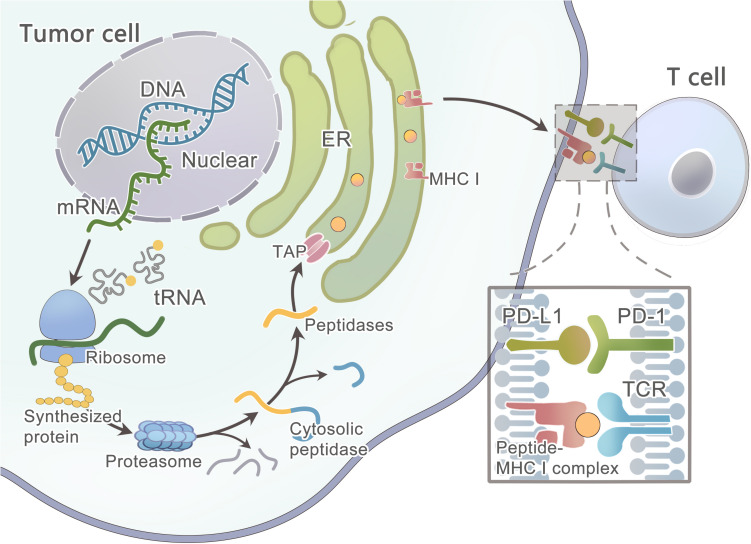
Schematic diagram illustrates the steps involved in tumor neoantigen processing and presentation on MHC class I molecules.

Primarily, the protein that contains the mutated residue is processed by the proteasome (Figure 3) (Nielsen et al., 2005) —a catalytic complex in the cytosol that can cleave the amino acid (AA) sequence to peptides ranging from 3 to 22 AA in length (Kisselev et al., 1999; Rock et al., 2002; Murata et al., 2009). A fraction of the peptides is further trimmed by aminopeptidases and endopeptidases in the cytosol and the endoplasmic reticulum(ER) (Beninga et al., 1998; York et al., 2002; Yan et al., 2006; Rock et al., 2010). Then, this peptide will be transported into the ER lumen by the TAP1/TAP2 transporter to assemble with MHC class I molecules (Peters et al., 2003; Larsen et al., 2007). Finally, the peptide-MHC class I complex can be presented on the cell surface. However, these computational tools hardly consider the endogenous processing and transport of peptides before HLA binding, which results in a high false-positive rate. As studied by Robbins and colleagues, 229 tumor-specific neo-epitopes were predicted in three melanoma patients, but only 11 (4.8%) of these neo-epitopes elicited a T cell response (Robbins et al., 2013). Neo-epitopes can also be produced by an altered MHC class I processing machinery in cancer cells (Van Hall et al., 2006; Seidel et al., 2012; Van Der Burg et al., 2016), which results in a high false-negative rate. Recently, Abelin et al. (2017) developed a new method of liquid chromatography-tandem mass spectrometry (LC–MS/MS) analysis of HLA-associated peptides which takes into account the endogenous processing of peptides, they also developed a new predictor with better algorithm since it is trained on peptide affinity data.

However, some patients may have no expression or aberrated expression of MHC molecules as immune evasion mechanisms (Marincola et al., 2000; Garrido et al., 2010; Leone et al., 2013; Textor et al., 2017). The loss of MHC class I can be detected in the early stage of some cancers (Vermeulen et al., 2005; Van Esch et al., 2014). As studied by Cabrera et al. (2003), β2m mutations and LMP7/TAP2 downregulation are the two main mechanisms in colorectal cancer that are responsible for the loss of MHC surface expression, and thus T cells fail to recognize cancer cells during an immune response. In some tumors, the MAPK pathway may regulate MHC I presentation (Mimura et al., 2013; Bradley et al., 2015; Ebert et al., 2016). After the mutated peptide and MHC class I complex presenting on the tumor cell surface, T cell recognition can occur only when TCRs that have the ability to recognize the mutant epitope exist within the T cell repertoire. Luckily, prior data showed that the T cell repertoire had a diversity of ∼2.5 × 10^7^ (Arstila et al., 1999), and a single TCR was able to recognize up to 10^6^ different MHC/peptide complexes, and thus the immune system could recognize ∼10^12^ possible foreign epitopes (Mason, 1998), which means the immune system has a strong recognition ability to distinguish even minor variations in MHC/peptide complexes. A study has shown that only certain types of mutations can be missed, such as conservative substitutions at other positions or alterations at the N-terminal peptide residue (Kessels et al., 2004).

### Tumor Heterogeneity

Genomic instability and mutational processes can result in extensive tumor heterogeneity in each patient (Gerlinger et al., 2012). First, spontaneous mutations occur during the stages of tumor progression. Second, tumor microenvironments such as T cells can mediate neoantigen immunoediting (Verdegaal et al., 2016) or neoantigen loss. Third, metastatic lesions can involve the distal outgrowth of tumor cells originated from a subclone of the primary tumor; although there is little heterogeneity in driver mutations, there is still considerable epigenetic reprogramming between primary and metastatic tumors as studied in pancreatic ductal adenocarcinoma (PDAC) (Alderton, 2017). Therefore, a single site tumor biopsy may not adequately capture the total number of antigen clones present in the tumors (Sankin et al., 2014). It is well established that intratumor heterogeneity (ITH) correlates with the response of cancer patients to treatments with targeted therapies (Diaz et al., 2012; Shi et al., 2014; Landau et al., 2015). This is only tumor heterogeneity for each individual patient. For different patients and different cancers, the neo-epitopes were rarely shared except for driver mutations, which account for a fraction of mutations. Charoentong et al. (2017) analyzed more than 8,000 patients comprising 20 solid cancers from the TCGA (results available at https://tcia.at/). The pan-cancer analysis showed that the fraction of neo-epitopes generated from driver genes was 7.6%. Only 24 of 911,548 unique predicted neo-epitopes were common in more than 5% of patients (Charoentong et al., 2017). Therefore, neoantigen immunotherapy will probably need to be fully personalized for each patient, and this will be the next generation of precision medicine.

### Tumor Suppression Environment

Cancer immunotherapy can offer limited clinical benefit without mitigating the immunosuppressive microenvironment of tumors. It has been increasingly recognized that tumors develop a specialized niche termed the tumor microenvironment (TME) in which tumor cells are protected from therapeutic interventions (Quail and Joyce, 2013). This niche includes fibroblasts (Kalluri and Zeisberg, 2006; Chen and Song, 2018), myeloid suppressor cells (MDSCs) (Goedegebuure et al., 2011; Kumar et al., 2016), regulatory T (Treg) cells (Van Der Burg et al., 2007; Bonertz et al., 2009), tumor-associated macrophages (TAM) (Qian and Pollard, 2010; Panni et al., 2013), lymphocytes, the extracellular matrix (ECM) and abnormal blood and lymphatic vessels (Jain, 2013). For example, high stromal density can limit T cell access to tumor cells and delivery of cytotoxic agents that provide a barrier (Provenzano et al., 2012; Feig et al., 2013; Ozdemir et al., 2014). Cancer cells can also express ligands for inhibitory receptors on T cells and secrete a multitude of chemokines and cytokines to affect antitumor immunity (Walker et al., 2003; Thomas and Massague, 2005). The vaccine strategies can successfully increase the frequency and activity of T cells, but they fail to guarantee that these T cells can exert their function within the tumors. The most important reason is the immune escape mechanisms in cancers, and thus proper co-treatment during vaccination is needed (Arens et al., 2013; Van Der Burg et al., 2016). Other than ICIs, there are multiple inhibitors targeting tumor immunosuppressive factors, including IDO1 inhibitors (Spranger et al., 2014), MEK inhibitors (Ebert et al., 2016), colony-stimulating factor-1 receptor (CSF1R) and chemokine (C-C motif) receptor 2 (CCR2) inhibitors (Mitchem et al., 2013), tumor extracellular matrix and stromal inhibitors (Provenzano et al., 2012), adenosine signaling through the adenosine A2a receptor (A2aR) (Leone et al., 2015), and other metabolic signaling pathways (Pardoll, 2015). In addition, combining such TME modulators with neoantigen-specific therapy may augment antitumor immunity (Melief et al., 2015). As studied by Zhu et al. (2014) CSF1R blockade could significantly improve the efficacy of PD-1 or CTLA-4 antagonists on tumor regressions. Furthermore, among the large number of predicted epitopes, only a minority can be recognized by autologous T cells (Robbins et al., 2013; Van Rooij et al., 2013; Rizvi et al., 2015); therefore, one group has proved that T cells redirected with T cell repertoires of healthy blood donors can efficiently recognize cancer epitopes that are neglected by a patient’s autologous T cells (Stronen et al., 2016).

## Conclusion and Perspectives

Personal neoantigen vaccines can elicite strong T cell responses, which not only expand existing neoantigen-specific T cell populations, but also induce a new proportion of specific T cells in cancer patients (Ott et al., 2017). Hence, the identification of neoantigens is of utmost importance to improve cancer immunotherapy and broaden its efficacy to a larger number of patients. Khodadoust et al. (2017) discovered that immunoglobulin neoantigens in human mantle-cell lymphomas and CD4+ T cells specific for the neoantigens could mediate killing of autologous lymphoma cells. Keskin et al. (2019) also demonstrated that a strategy using multi-epitope, personalized neoantigen vaccination is feasible not only for high-risk melanoma (Rizvi et al., 2015; Ott et al., 2017; Sahin et al., 2017) but also for glioblastoma (Keskin et al., 2019), which has a relatively low mutation load. Furthermore, Balachandran et al. (2017) identified MUC16 as immunogenic hotspots in long-term survivors of pancreatic ductal adenocarcinoma, which is a presumed poorly immunogenic and checkpoint blockade-refractory tumor. These results are encouraging, but efforts are needed to tackle those challenges, and we will be very likely to witness these exciting developments in the near future.

## Author Contributions

X-JH and XM was a major writer of the manuscript and designed the figures, tables. XW and YW developed the structure of the article, reviewed and edited the manuscript. LY and YP researched the appropriate references and reviewed the manuscript. All the authors read and approved the final manuscript.

## Conflict of Interest

The authors declare that the research was conducted in the absence of any commercial or financial relationships that could be construed as a potential conflict of interest.

## Footnotes

1 http://cancer.sanger.ac.uk

2 http://www.cancersplicingqtl-hust.com/

3 http://srv00.recas.ba.infn.it/atlas/

4 http://RNAedit.com
